# Hypothalamic Nesfatin-1 Resistance May Underlie the Development of Type 2 Diabetes Mellitus in Maternally Undernourished Non-obese Rats

**DOI:** 10.3389/fnins.2022.828571

**Published:** 2022-03-21

**Authors:** Máté Durst, Katalin Könczöl, Klementina Ocskay, Klaudia Sípos, Péter Várnai, Anett Szilvásy-Szabó, Csaba Fekete, Zsuzsanna E. Tóth

**Affiliations:** ^1^Laboratory of Neuroendocrinology and in situ Hybridization, Department of Anatomy, Histology and Embryology, Semmelweis University, Budapest, Hungary; ^2^Department of Physiology, Semmelweis University, Budapest, Hungary; ^3^Laboratory of Integrative Neuroendocrinology, Institute of Experimental Medicine, Budapest, Hungary

**Keywords:** IP-ITT, IP-GTT, neuronal birth dating, cFos, arcuate nucleus, nesfatin-1

## Abstract

Intrauterine growth retardation (IUGR) poses a high risk for developing late-onset, non-obese type 2 diabetes (T2DM). The exact mechanism underlying this phenomenon is unknown, although the contribution of the central nervous system is recognized. The main hypothalamic nuclei involved in the homeostatic regulation express nesfatin-1, an anorexigenic neuropeptide and identified regulator of blood glucose level. Using intrauterine protein restricted rat model (PR) of IUGR, we investigated, whether IUGR alters the function of nesfatin-1. We show that PR rats develop fat preference and impaired glucose homeostasis by adulthood, while the body composition and caloric intake of normal nourished (NN) and PR rats are similar. Plasma nesfatin-1 levels are unaffected by IUGR in both neonates and adults, but pro-nesfatin-1 mRNA expression is upregulated in the hypothalamus of adult PR animals. We find that centrally injected nesfatin-1 inhibits the fasting induced neuronal activation in the hypothalamic arcuate nucleus in adult NN rats. This effect of nesfatin-1 is not seen in PR rats. The anorexigenic effect of centrally injected nesfatin-1 is also reduced in adult PR rats. Moreover, chronic central nesfatin-1 administration improves the glucose tolerance and insulin sensitivity in NN rats but not in PR animals. Birth dating of nesfatin-1 cells by bromodeoxyuridine (BrDU) reveals that formation of nesfatin-1 cells in the hypothalamus of PR rats is disturbed. Our results suggest that adult PR rats acquire hypothalamic nesfatin-1-resistance, probably due to the altered development of the hypothalamic nesfatin-1 cells. Hypothalamic nesfatin-1-resistance, in turn, may contribute to the development of non-obese type T2DM.

## Introduction

Type 2 diabetes (T2DM) is a global health concern. Besides causing serious complications, T2DM has recently been associated with increased odds of in-hospital death with COVID-19 (Barron et al., 2020). Although obesity is a major risk factor for T2DM, relatively large proportion of T2DM patients (around 20% in Europe, and much higher in Asia) are non-obese (Vaag and Lund, 2007), and the disease develops independently of the amount of the adipose tissue hormone, leptin (Wang et al., 2014). The etiology and pathophysiology of non-obese T2DM are unclear. However, intrauterine growth retardation (IUGR) combined with low birthweight is a primary contributor to the development of the disease (Vaag and Lund, 2007). This condition is usually attributed to a poor intrauterine environment, one of the most common complications of pregnancy (Saleem et al., 2011).

Intrauterine undernourishment induced by protein restriction (PR) is a widely used rodent model in T2DM research (Martin-Gronert and Ozanne, 2007; Pinney, 2013). This model reproduces well the consequences of human IUGR (Saleem et al., 2011). The adverse fetal environment induces a predictive adaptive response (fetal programing) in the organism (Barker et al., 1993), and permanently alters the function of the hypothalamic circuits regulating food intake, energy expenditure and glucose homeostasis. Metabolic syndrome develops depending on the genetic predisposition of the individual and the quality of the postnatal environment (Levin, 2006; Carey et al., 2013; Pedroso et al., 2017; Timper and Brüning, 2017; Durst et al., 2019).

Nesfatin-1, the N-terminal fragment of the nucleobindin-2 protein (NUCB2) is an anorexigenic neuropeptide (Oh-I et al., 2006; Maejima et al., 2009; Shimizu et al., 2009), although its production has also been demonstrated in several peripheral organs (Wei et al., 2018). It is highly expressed in the arcuate (ARC), paraventricular (PVN), and supraoptic (SON) nuclei of the hypothalamus and in the lateral hypothalamic area (LHA) (Brailoiu et al., 2007; Foo et al., 2008). All these areas are considered hypothalamic feeding centers (Schwartz et al., 2000; Maejima et al., 2018). Acute intracerebroventricular (icv) administration of nesfatin-1 reduces food intake and increases energy expenditure in a leptin independent way (Oh-I et al., 2006; Könczöl et al., 2012). Chronic nesfatin-1 treatment leads to weight loss (Oh-I et al., 2006).

Nesfatin-1 is also an important factor in the central control of glucose homeostasis. Autonomic control of the pancreas and the liver arises mainly from the hypothalamic ARC and PVN nuclei as well as the LHA (Uyama et al., 2004; Sandoval et al., 2009; Pozo and Claret, 2018), which contain a high density of nesfatin-1 neurons. Insulin sensitivity in the liver and peripheral tissues increases upon icv infusion of nesfatin-1 in rats during euglycemic-hyperinsulinemic clamps (Guo et al., 2013). Nesfatin-1 delivered into the hypothalamus *via* the third ventricle inhibits hepatic glucose production and promotes glucose uptake in skeletal muscle (Gantulga et al., 2012; Yang et al., 2012; Guo et al., 2013; Nakata and Yada, 2013). Based on collective data, NUCB2/nesfatin-1 with its associated regulatory processes are recognized as promising targets for treating type 2 diabetes, obesity and metabolic syndrome (Nakata and Yada, 2013).

Considering that (1) IUGR is one of the main risk factors for developing non-obese T2DM, (2) fetal programing of the central nervous system contributes primarily to the development of the IUGR phenotype, (3) the role of hypothalamic NUCB2/nesfatin-1 in the regulation of food intake and glucose homeostasis is established and that (4) nesfatin-1 acts in a leptin independent manner, the role of NUCB2/nesfatin-1 in the development of the non-obese type T2DM in the IUGR phenotype can be assumed. To investigate this, we used a PR rat model of IUGR and characterized the development, food preference, glucose homeostasis as well as the hypothalamic nesfatin-1 system of these animals. We also examined the effect of acute and chronic nesfatin-1 treatment on glucose homeostasis in our model.

## Materials and Methods

### Animals and Tissue Handling

Rats (Wistar, Toxi-Coop Toxicological Research Center Zrt, Dunakeszi, Hungary) were housed under standard laboratory conditions (22 ± 1°C, 12-h day cycle) and had free access to food and water. Experiments were performed using males, except otherwise indicated. During brain surgeries and transcardial perfusions, anesthesia was applied using a mixture of ketamine (75 mg/bwkg) (Richter Gedeon Nyrt, Budapest, Hungary) and xylazine (15 mg/bwkg) (CP-Pharma, Burgdorf, Germany) given intramuscularly. Animals were handled daily for two weeks before experiments to avoid experimental stress. Tissues were stored at −80°C until used.

Experiments conformed to the European Communities Council Directive (2010/63/EU) and were approved by the Semmelweis University Employment and Animal Welfare Committee (XIV-I-001/2262-4/2012). The approval numbers of the Research Ethics Committee are PE/EA/1563-7/2017, PE/EA/1122-2-7/2020.

### PR Rat Model

PR and normal nourished (NN) rats were generated as we earlier described (Durst et al., 2019). Briefly, the cycle of the females was monitored by daily examination of the vaginal smear. Females were selected for mating in proestrus or estrus. Females were considered timed-pregnant, if sperm cells appeared in the vaginal smear the following morning. This day was considered the embryonic day zero (E0). Pregnant female rats were then housed individually and kept either on a standard diet (Maintenance diet, Altromin Spezialfutter GmbH, Lage, Germany, catalog# 1324), or a low protein (42% protein relative to standard) diet (Protein deficient diet I, Altromin Spezialfutter GmbH, Lage, Germany, catalog# C1003) during pregnancy (Table 1). After delivery, all dams received standard rat chow (Desai et al., 2007) and litter sizes were adjusted to eight male pups. Bodyweight (bw) of the offsprings were measured weekly. To avoid the confounding effects of any inter-litter differences or bias subjects of three different litters were selected into each experimental group. Experiments were performed using neonates (maximum 4 days old), 6 weeks old and adult, 12 weeks old animals.

**TABLE 1:** Composition of the diets used in the study.

	Standard diet		High fat diet		Low protein diet	
	(g%)	(kcal %)	(g%)	(kcal %)	(g%)	(kcal %)
Protein	19.2	23.9	12.8	10	8.1	9.2
Carbohydrate	53.4	64.8	35.6	27.3	70.5	78
Fat	4.1	11.3	36.07	62.7	5.1	12.8
Energy (kcal/g)	3.23		5.35		3.56	

### Magnetic Resonance Imaging

Body-composition analysis (fat content, lean body weight, free and total water content) was performed in adult (12-week-old), awake NN and PR rats (*n* = 5/group) using an EchoMRI 700 Whole Body Composition Analyzer (Zinsser Analytic GmbH, Frankfurt am Main, Germany).

### Food Preference Test

Fat preference of NN and PR rats (*n* = 6/group) was tested at 6 and 12 weeks of age. Rats were kept individually, and offered by both standard and high fat diet (HFD) on an *ad libitum* basis for 10 days. The diet of each rat was measured manually at the beginning and at the end of the experiment and the individual food consumption was calculated. HFD contained standard rat chow supplemented with 50% extra lard (Table 1) (Tesco, Budaörs, Hungary) (Polyak et al., 2016). The left and right positions of the diets in the hoppers of the cages were changed daily. The food preference test of 12-week-old animals was repeated using another cohort of rats to obtain daily data. The caloric intake/bwg values were calculated and used for statistical analyses.

### Intraperitoneal Glucose (IP-GTT) and Insulin (IP-ITT) Tolerance Tests

IP-GTT was performed to assess the ability of animals to metabolize glucose. IP-ITT was performed to assess the sensitivity of insulin-responsive tissues. We used untreated 6 week-old and adult NN and PR rats (*n* = 8/group), as well as adults with previous chronic nesfatin-1 or vehicle treatment (see below). After overnight fasting, rats were given ip injections of 2 g/bwkg glucose (Merck Kft., Budapest, Hungary) or 0.75 IU/bwkg insulin (Humulin R, Eli Lilly, Utrecht, Netherlands) diluted in saline. Glucose concentration was measured in blood samples collected from the tip of the tail just before (0 min), as well as 15, 30, 60, 90, 120, and 150 min after glucose (IP-GTT) or insulin (IP-ITT) challenges using a D-cont^®^ Trend Blood Glucose Meter (77 Elektronika Kft., Budapest, HU).

### Birth Dating of Hypothalamic Cells by Bromodeoxyuridine Labeling

Timed-pregnant rats were given a single dose (160 mg/bw kg) of intraperitoneal (ip) bromodeoxyuridine (BrDU) (Merck) injection on the gestational day 13 (Markakis, 2002). NN and PR male offsprings (*n* = 4/group) of different dams were perfusion-fixed with 4% paraformaldehyde dissolved in PBS (Merck) at 12 weeks of age. The brains were removed and processed for BrDU and nesfatin-1 immunohistochemistry (see below). Two animals/group was omitted from the colocalization study, as they failed to show any BrDU labeling in the brain.

### Acute Intracerebroventricular Nesfatin-1 Administrations

Cannulation and nesfatin-1 administration was performed as described earlier (Konczol et al., 2010). Briefly, a polyethylene guide cannula (Smiths Medical ASD, Inc., NH, United States) was inserted stereotaxically into the right lateral ventricle (0.8 mm caudal to the bregma, 2.0 mm lateral to the sagittal suture and 4.0 mm below the skull surface) of adult rats. The placement of the cannulas was verified by injecting 10 nM/3 μl of angiotensin II into the brains 1 day after surgery. Only animals reacting with an intensive drinking response were included in the study. Following surgery, rats were kept individually and allowed to recover for one week. On the day of the experiment, the rats were randomly divided into four groups: NN-Nesfatin-1, PR-Nesfatin-1, NN-Saline, PR-Saline. Nesfatin-1 (Phoenix Pharmaceuticals Inc., Burlingame, CA, United States) was dissolved in saline at a concentration of 5 pmol/μl. Each rat received 5 μl of nesfatin-1 (25 pmol), or saline icv at the beginning of the dark cycle. Two different experiments were performed.

1. To measure the effect of nesfatin-1 on food and water intakes, food was withdrawn from the animals (*n* = 5/group) 1 h before the injections. Immediately after the injections (zero timepoint) food was returned to rats and their food and water intakes were measured manually at certain intervals of time (0–4, 4–8, and 8–12 h).
2. To understand the effect of nesfatin-1 on fasting-induced neuronal activation, the animals (*n* = 7/group) were fasted for 24 h. Rats were perfusion-fixed with 4% paraformaldehyde 90 min after the icv injections. The brains were removed and processed for cFos immunohistochemistry (see below).

### Chronic Intracerebroventricular Nesfatin-1 Treatment

A catheter (Alzet^®^ brain-infusion kit 2) connected to an osmotic minipump (Alzet^®^ Model 2001, Durect Corporation, Cupertino, CA, United States) was implanted into the right cerebral ventricle of rats according to the manufacturer’s instructions. The minipump delivered nesfatin-1 (70 pmol/day) or vehicle [sterile artificial cerebrospinal fluid (ACSF), composition in mmol/l: NaCl: 140, KCl: 3.35, MgCl_2_: 1.15, CaCl_2_: 1.26, Na_2_HPO_4_:1.2, NaH_2_PO_4_: 0.3] icv to rats with 1 μl/h pumping rate for 7 days (cohort 1: NN-nesfatin-1 and NN-aCSF, *n* = 6/group; cohort 2: PR-nesfatin-1 and PR-aCSF, *n* = 6/group). Food intake and bodyweight of rats were measured daily. At the end of the infusion period, rats were tested for glucose and insulin tolerance (see below). After the experiments the animals were sacrificed by decapitation. The accuracy of the placement of the cannulas was verified later histologically.

### Quantitative *in situ* Hybridization

NUCB2 mRNA levels in the different hypothalamic nuclei were determined by radioactive *in situ* hybridization (ISH) providing a linear relationship between the signal intensity and the mRNA expression level (Chen C. C. et al., 2012). Hybridizations were performed using fresh frozen coronal sections as earlier described by [^35^S]UTP-labeled (Per-Form Hungária Kft, Budapest, Hungary) antisense riboprobes (Tóth et al., 2008; Konczol et al., 2010). Riboprobes were generated by *in vitro* transcription according to the manufacturer instructions (MAXIscript Kit, Thermo Fisher Scientific, Budapest, Hungary). The cDNA template for the *in vitro* transcription corresponded to the 286–531 bs of the rat NUCB2 sequences. Specificity of cDNA was verified by sequencing and assessed by BLAST screening^1^ of the rat genome. Fresh-frozen 12 μm thick serial coronal sections from the brains of NN and PR rats (neonates on postnatal day 0) (*n* = 4–6/group) and adults (*n* = 6/group) were hybridized overnight with 10^6^ DPM/slide of radioactively labeled riboprobe. Next day, after washing and dehydrating steps (Tóth et al., 2008; Konczol et al., 2010), sections were apposed to an imaging plate (Fuji Photo Film Co., Ltd., Kanagawa, Japan, NJ) for a week. Data were read out by a Fujifilm FLA-8000 Image Analyzer. Optical densities (mean gray values) of the individual nuclei were measured from the storage phosphor recordings with the help of the ImageJ 1.32j software (Wayne Rasband; NIH, Bethesda, MD, United States). The same nuclei were evaluated by applying the same settings both across the sections and across the animals. Background values were measured in parallel and subtracted. Data were assessed bilaterally from at least two sections per animal selected from the centers of the investigated areas (SON, PVN, LHA, ARC). The average/animal data were compared statistically.

### Enzyme-Linked Immunosorbent Assay

Peripheral nesfatin-1 protein levels were determined from plasma samples of PR and NN neonates (4-day old) and adults (12-week-old) using a Nesfatin-1 (1–82) (Rat) EIA kit (EK-003-22) (Phoenix) according to the manufacturer’s instructions (*n* = 3/group). All samples were measured in one assay, the intraassay coefficient of variability was 2.52.

### Immunohistochemistry

The perfusion-fixed brains were cryoprotected in 20% sucrose (Merck) solution and then frozen in dry ice-cold methyl-butane (Merck). Free-floating 50 μm thick serial coronal sections of the hypothalamus were cut by a frigomobil (Frigomobil, Reichter-Jung, Vienna, Austria). Immunohistochemistry was performed using standard protocols. For cFos immunostaining we used a rabbit anti-cFos (1:20,000, Cat. # ABE 457, Merck) primary antibody. The cFos signal was developed by using the standard ABC method and nickel-diaminobenzidine as chromogen. For BrDU and nesfatin-1 double fluorescence immunostaining, the sections were treated with 2N HCl at 37°C for 30 min to perform antigen retrieval. The following primary antibodies were used: mouse anti-BrDU (1:1,000, Cat. # B8434, Merck) and rabbit anti-nesfatin-1 (1:6,000, Cat. # H-003-22, Phoenix, recognizes also NUCB2 protein). The BrDU and nesfatin-1 immunostainings were visualized sequentially by using TSA Kits containing Alexa Fluor 488 and 568 fluorophores, respectively (Cat. # T20912, T20924, Thermo Fisher Scientific). Microwave treatment in 0.1 M citric acid at pH 6 was used to eliminate the activity of the secondary antibody conjugated peroxidase enzyme between the two detection steps (Toth and Mezey, 2007).

### Cell Counting

The number of cFos-labeled cells was counted in light microscopic pictures (see imaging) within regions of interests (ROIs). ROIs were placed within the centers of the relevant hypothalamic nuclei at the same rostro-caudal level across the animals determined by using the Rat Brain Atlas (Paxinos and Watson, 2007). The size of the ROI was 100 × 100 μm (SON, parvocellular PVN, magnocellular PVN, ARC), or 200 × 200 μm (LHA). The SON and subnuclei of PVN were evaluated using one ROI/nucleus. Two and three ROIs were placed over different regions of the ARC and LHA, respectively. Data of 4 (SON, PVN) or 7 (ARC, LHA) sections per animal were assessed bilaterally with the help of the cell counter tool (manual counting) in the ImageJ application. The cells were counted by a person skilled in the evaluation of immunohistochemical stainings. The average count per ROI/animal data were compared statistically.

The number of nesfatin-1 cells in adults (*n* = 4/group) and the BrDU and nesfatin-1 double immunostaining (*n* = 2/group) was evaluated in two non-consecutive sections at 2.9 mm and 3.1 mm caudal to the bregma. The hypothalami were outlined on tile-scanned confocal images (see imaging) with the help of the Rat Brain Atlas (Paxinos and Watson, 2007). Nesfatin-1^+^ and BrDU^+^ cells in the different channels were labeled and counted bilaterally with the help of the ImageJ program. The number of nesfatin-1 cells/section/animal and the percentage of BrDU^+^ nesfatin-1 cells/section/animal were compared statistically.

### Imaging

Light microscopic pictures of cFos immunostained sections were taken by an Olympus BX60 microscope (objectives: UPlanFLN 10×/0.30) interfaced with a SPOT Xplorer 17.4 camera (Diagnostic Instruments Inc., Sterling Heights, MI, United States). Confocal tile scans of BrDU and nesfatin-1 double-labeled sections were acquired on a LSM 780 confocal laser-scanning microscope (Zeiss, Jena, Germany) using a 20×/0.8 M27 objective with a resolution of 2.4 pixel/micron and pinhole diameter 0.00057. The images were stitched using the ZEN 2010 program. Contrast and sharpness of the illustrative images were adjusted in Adobe Photoshop CS 8.0. Multi-panel figures were assembled in PowerPoint 2016.

### Statistics

Data analyses were performed by investigators blinded to experimental groups. The size of the experimental groups was determined based our previous experience (Könczöl et al., 2012; Durst et al., 2019). Statistical significances were calculated employing the Sigmastat 3.5 application (Systat Software Inc., Chicago, IL). Student’s *t*-test (two-tailed) was used for comparing two groups with normal distribution of data, otherwise Mann-Whitney *U-*test was applied. When comparing the effects of two factors, two-way ANOVA, or for consecutive measurements two-way RM ANOVA was applied followed by Tukey *post hoc* analysis. The statistics were performed using the original data values. Differences between groups were considered statistically significant, when *p* < 0.05. Results are expressed as means ± SEM values.

## Results

### PR Animals Exhibit Fast Catch-Up Growth, Enhanced Fat Preference and Impaired Glucose Homeostasis

**FIGURE 1:**
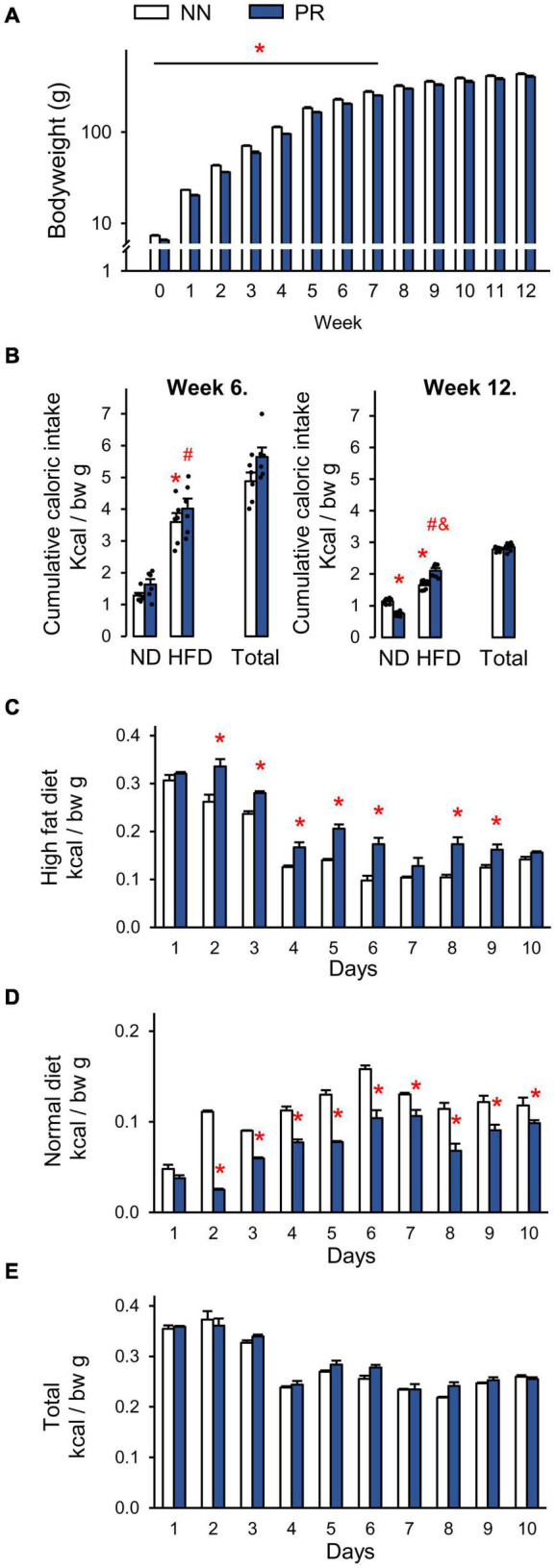
Postnatal development of normal nourished (NN) and intrauterine protein restricted (PR) rats as well as relative caloric intakes of normal (ND) and high fat (HFD) diets. **(A)** Weekly bodyweights. PR rats had lower than normal bodyweight at birth (week 0) and until week 8. Student’s *t*-test, or Mann-Whitney *U*-test, NN vs. PR *p < 0.05, *n* = 6. Note the logarithmic scale on the y axis. **(B)** Caloric intakes of ND and HFD. Both diets were offered for 10 days as a free choice. Both NN and PR rats prefer HFD over ND at postnatal week 6 (left, catch up growth period) and postnatal week 12 (right, catch up growth completed). At postnatal week 12 PR rats consume relatively more of HFD and less of ND than NN rats. The total caloric intake of PR rats is normal. ND—HFD bars: Two-way ANOVA, week 6: effect of diet: *F*_(1_, _20)_ = 101.87, *p* < 0.001, week 12: phenotype × diet: *F*_(1_, _20)_ = 49.01, *p* < 0.001. Tukey multiple comparison test, *p < 0.001 vs. NN-ND, ^#^*p* < 0.001 vs. PR-ND, ^&^*p* < 0.001 vs. NN-HFD. **(C–E)** Relative daily caloric intakes during the fat preference test performed at postnatal week 12. **(C)** HFD. Two-way RM ANOVA, effect of time: *F*_(9_, _90)_ = 286.90, *p* < 0.001, effect of phenotype: *F*_(1_, _90)_ = 19,28, *p* < 0.001, phenotype × time: *F*_(9_, _90)_ = 8.48, *p* < 0.001. **(D)** ND. Two-way RM ANOVA, effect of time: *F*_(9_, _90)_ = 107.81, *p* < 0.001, effect of phenotype: *F*_(1_, _90)_ = 51.06, *p* < 0.001, phenotype × time: *F*_(9_, _90)_ = 20.03, *p* < 0.001. **(E)** HFD + ND as total. Two-way RM ANOVA, effect of time: *F*_(9_, _90)_ = 168.83, *p* < 0.001. Tukey multiple comparison tests, NN vs. PR within day *p < 0.01. Means ± SEM, *n* = 6 for all graphs. bw: bodyweight.

PR animals were born with a lower body weight than NN animals and this difference was maintained until the eighth week after birth. This time can be considered the end of the catch-up growth period (Figure 1A). From week 8 until the end of the experiment (week 12), the bodyweights of the PR and NN rats were similar. Analysis of body composition by EchoMRI also confirmed that the fat content, lean and total body masses, as well as total and free water content of PR rats at 12 weeks of age were similar to those of NN rats (Table 2).

**TABLE 2:** Body composition of the adult (12 week-old) experimental animals.

	Total body mass (g)	Fat content (%)	Lean body mass (%)	Total water content (%)	Free water content (%)
Control	446 ±17	14.3 ± 0.69	80.2 ± 0.73	67.0 ± 0.70	0.32 ± 0.02
PR	494 ± 9	16.3 ± 0.94	77.5 ± 0.95	64.6 ± 0.70	0.34 ± 0.04

*p*	0.176	0.251	0.232	0.24	0.591

*Mean ± SEM as well as p-values calculated by Student’s t-test, n = 5.*

In another experiment, we measured the caloric intake of rats and examined their fat preference. During the catch-up growth period, at 6 weeks of age (Figure 1B, left), rats in both groups showed a similar preference for HFD over ND. The total caloric intake per bodyweight was therefore not different between the groups (Figure 1B, left). After the catch-up growth period, at 12 weeks of age, rats continued to prefer HFD over ND, but to a different extent depending on phenotype (Figure 1B, right). PR rats consumed more calories from HFD, than NN rats. Since they consumed correspondingly less calories from ND, the total caloric intake per bodyweight remained the same in the two groups (Figure 1B right).

Daily analysis of the preference test of adults revealed that calorie consumption from HFD was initially very high (Figure 1C). It then decreased over time in both groups, and remained more or less constant after day 6. The increased fat preference of PR rats relative to controls was observed from day 2 and lasted practically until the end of the observation period (Figure 1C). Calorie consumption of ND showed an opposite dynamics (Figure 1D). It increased over time in both groups until about day 6, and did not change dramatically thereafter. From day 2 of the testing period, PR rats consumed fewer calories from ND, than NN rats (Figure 1D). The caloric intake from HFD was extremely high in both groups at the beginning of the testing period, and no compensation was made at the expense of ND. Therefore, the daily total caloric intakes were higher on the first 3 days compared to the other days (Figure 1E). However, there was no difference between the phenotypes on any of the experimental days (Figure 1E).

**FIGURE 2:**
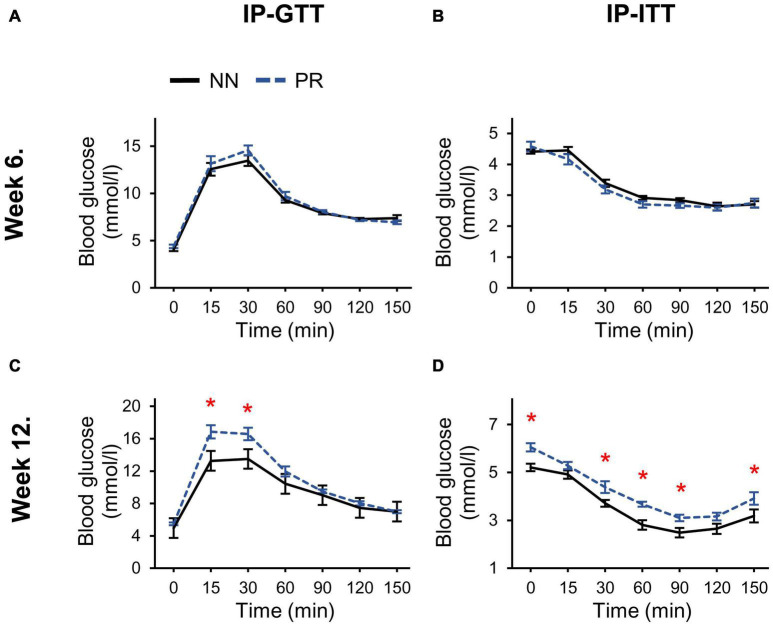
Glucose and insulin tolerances of NN and PR rats. **(A)** Intraperitoneal glucose (IP-GTT) and **(B)** intraperitoneal insulin tolerance tests (IP-ITT) performed at postnatal week 6 show similar responses to glucose and insulin in both groups. **(C)** IP-GTT performed at postnatal week 12 shows decreased glucose tolerance of PR rats. Two-way RM ANOVA, effect of phenotype: *F*_(1_, _78)_ = 6.494, *p* = 0.023, effect of time: *F*_(6_, _78)_ = 87.342, *p* < 0.001, effect of phenotype × time: *F*_(6_, _78)_ = 2.972, *p* = 0.011. **(D)** IP-ITT shows decreased insulin sensitivity of PR rats compared to NN controls. Two-way RM ANOVA, effect of phenotype: *F*_(1_, _81)_ = 18.714, *p* < 0.001, effect of time: *F*_(6_, _81)_ = 93.725, *p* < 0.001. Tukey multiple comparison tests, NN vs. PR within one time point, *p < 0.05. Means ± SEM, *n* = 8 for all graphs.

The response to the intraperitoneal glucose and insulin treatment at week 6 was similar in PR and NN rats (Figures 2A,B). However, by week 12, both glucose tolerance and insulin sensitivity of PR rats were reduced (Figures 2C,D). In addition, fasting blood glucose levels (zero timepoint) of PR rats were elevated at week 12 (cohorts_1+2_ PR: 5.77 ± 0.14 mmol/l, NN: 5.09 ± 0.12 mmol/l, Student’s *t*-test, *p* < 0.001).

### Alterations in the Hypothalamic NUCB2/Nesfatin-1 System Characterize the PR Rats

**FIGURE 3:**
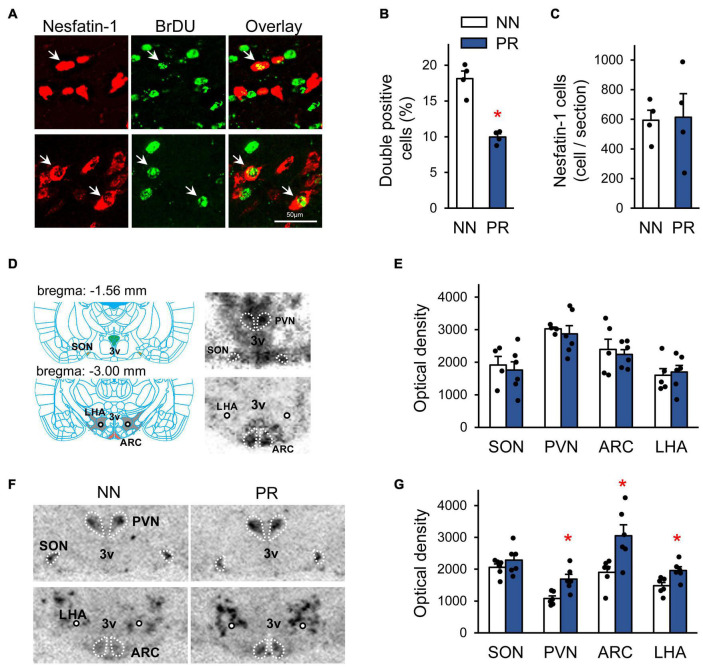
Characterization of the peripheral and central nesfatin-1 system. **(A)** Birth dating of nesfatin-1 neurons by bromodeoxyuridine (BrDU) at the embryonic day 13 (E13). Confocal microscopic images showing nesfatin-1 and BrDU double-labeled cells (arrows) in the hypothalamus of a NN rat. The immunostaining was performed using coronal hypothalamic sections of adult offspring. **(B)** Percentage of nesfatin-1 cells generated at E13 (BrDU and nesfatin-1 double-labeled cells) in the hypothalamus. Two-way RM-ANOVA, effect of phenotype *F*_(1_, _2)_ = 71.83, *p = 0.014, *n* = 2 animals and 4 sections/group. **(C)** The number of hypothalamic nesfatin-1 positive cells in adults. *n* = 4 animals/group. **(D)** Left: Schematic (Paxinos and Watson, 2007) showing the location of the investigated hypothalamic nuclei in coronal brain sections. The rostro-caudal distance from the bregma level is indicated for each section. ARC, arcuate nucleus; PVN, paraventricular nucleus; SON, supraoptic nucleus; LHA, lateral hypothalamic area; 3V, 3rd ventricle; circle, fornix. Right: Detection of NUCB2/nesfatin-1 mRNA by radioactive *in situ* hybridization technique in the investigated areas in neonatal rats. Autoradiographic images (NN rat) of coronal sections. The darker the shades of gray, the stronger the signal. **(E)** Bar graph showing NUCB2/nesfatin-1 mRNA levels in the investigated nuclei in neonates, *n* = 4–6. **(F)** Autoradiographic images showing NUCB2/nesfatin-1 mRNA expression in adult NN and PR rats. Note the enhanced signal intensity in most of the measured nuclei in the PR rat. **(G)** Bar graph showing NUCB2/nesfatin-1 mRNA levels in the investigated areas in adults. Student’s *t*-test, or Mann-Whitney *U*-test, NN vs. PR *p < 0.05, *n* = 6. Bar graphs show means ± SEM.

To assess whether fetal programming affected the development of hypothalamic nesfatin-1 neurons, we performed birth dating of cells by BrDU on the embryonic day 13 (E13) (Figure 3A). E13 was chosen based on data from previous studies (Altman and Bayer, 1986; Markakis, 2002). Our data suggest, that fewer nesfatin-1 cells were generated on E13 in PR rats than in NN rats (Figure 3B). However, the number of nesfatin-1 positive cells in adulthood was similar between groups (Figure 3C).

Next, we examined the expression of NUCB2/nesfatin-1 mRNA in the SON, PVN, ARC and LHA by ISH (Figure 3D). NUCB2/nesfatin-1 mRNA was detectable at birth in all investigated areas, and the expression levels in PR animals were not different from those observed in NN rats (Figures 3D,E). In contrast, in adult, 12 week-old PR rats NUCB2/nesfatin-1 mRNA expression was markedly upregulated in the PVN, ARC and LHA (Figures 3F,G). However, blood concentrations of nesfatin-1 were similar between groups in both neonates (5.0 ± 0.6 and 6.2 ± 1.0 ng/μl in NN and PR groups, respectively, Student’s *t*-test, *p* = 0.329, *n* = 3/group) and adults (11.1 ± 1.7 and 7.7 ± 0.7 ng/μl in NN and PR groups, respectively, Student’s *t*-test, *p* = 0.139, *n* = 3/group).

### PR Animals Develop Hypothalamic Nesfatin-1 Resistance by Adulthood

**FIGURE 4:**
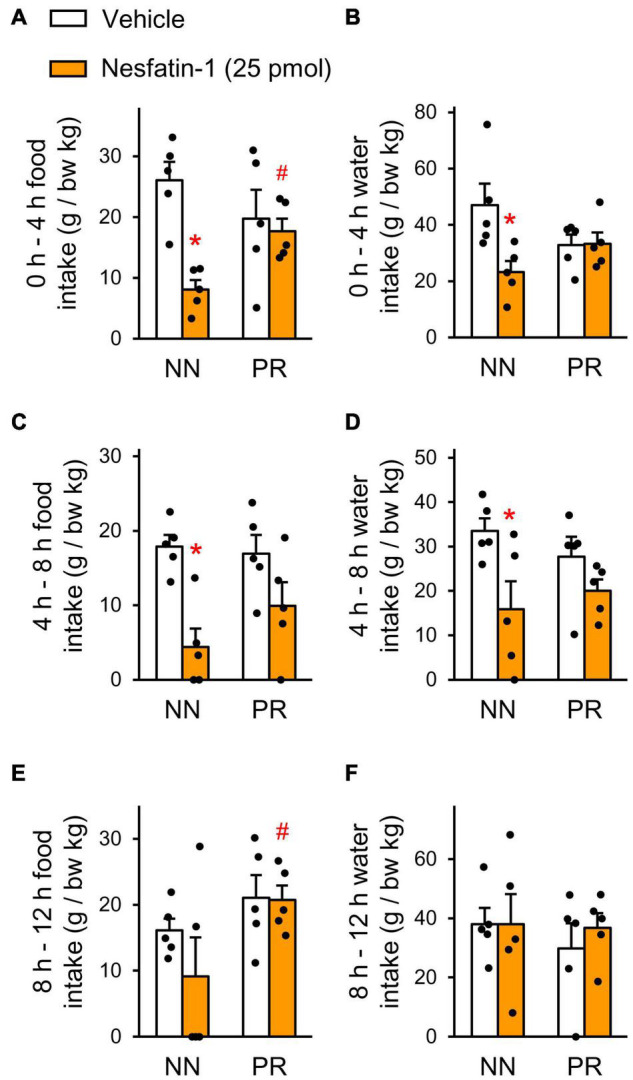
Effect of acute intracerebroventricular (icv) injection of nesfatin-1 (25 pmol), or vehicle on nocturnal food and water intakes of NN and PR rats. Food **(A,C,E)** and water **(B,D,F)** intakes of 12 week-old NN and PR rats were measured at 4 h intervals. Zero is the timepoint of treatments. **(A,B)** 0–4 h. Two-way ANOVA, food intake, effect of treatment *F*_(1_, _16)_ = 10.43, *p* = 0.005 treatment × phenotype: *F*_(1_, _16)_ = 6.530, *p* = 0.021, water intake, treatment × phenotype: *F*_(1_, _16)_ = 5.66, *p* = 0.030. **(C,D)** 4–8 h. Two-way ANOVA food intake, effect of treatment: *F*_(1_, _16)_ = 16.85, *p* < 0.001, water intake, effect of treatment: *F*_(1_, _16)_ = 8.51, *p* < 0.010. **(E,F)** 8–12 h. Two-way ANOVA, effect of phenotype: *F*_(1_, _16)_ = 5.04, *p* = 0.039; NN vs. PR within nesfatin-1, *p* = 0.041. Effects of nesfatin-1 are impaired in PR rats. Tukey multiple comparison tests, *p < 0.05 vs. NN-vehicle, ^#^*p* < 0.05 vs. NN-nesfatin-1. Means ± SEM, *n* = 5 for all graphs.

To investigate the functional significance of the alterations found in the hypothalamic nesfatin-1 system of PR rats, we administered nesfatin-1 icv to the rats and measured their food and water intakes. Nesfatin-1 significantly reduced the relative food and water intake of NN rats in the first 4 h after treatment (Figures 4A,B). Food and water consumption of the PR group was not affected by nesfatin-1 during this period. Similar observations were made between 4 and 8 h post-treatment (Figures 4C,D). The effect of nesfatin-1 on the food intake continued to differ between NN and PR rats for the remainder of the dark phase (Figure 4E). Water consumption was no longer affected by nesfatin-1 during this period (Figure 4F).

**FIGURE 5:**
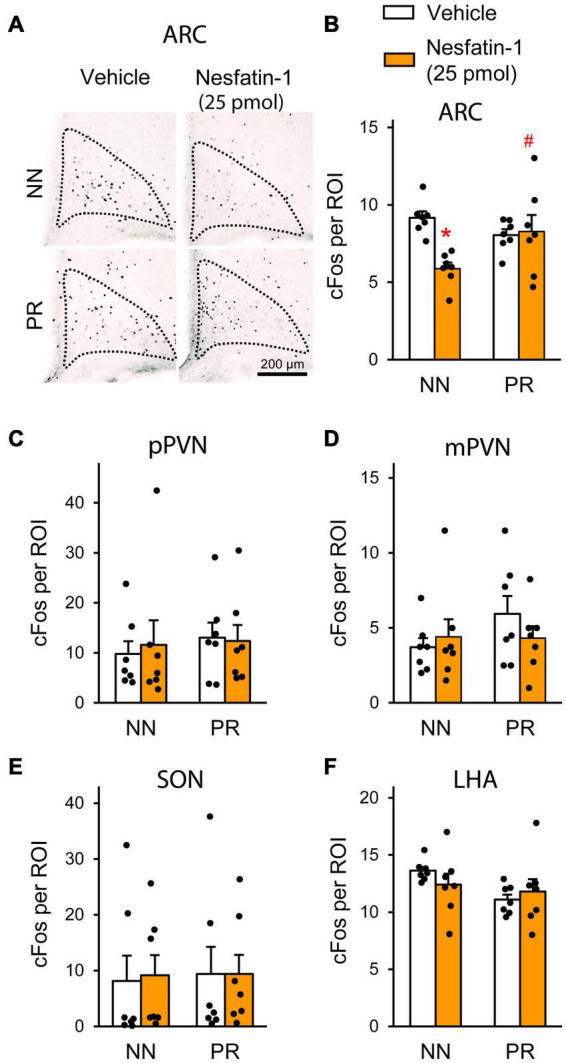
Neuronal activation (cFos) in the hypothalamus following acute icv nesfatin-1 injection (25 pmol) into NN and PR rats subjected to overnight fasting. **(A)** Representative light microscopic images showing cFos-immunoreactive cell nuclei (black dots) mainly within the medial ARC of the different experimental groups. **(B–F)** Bar graphs showing the quantitative data on the number of cFos-positive cells per region of interest (ROI). **(B)** ARC. Nesfatin-1 reduced the number of fasting activated neurons in the NN rats. Two-way ANOVA, effect of treatment: *F*_(1_, _24)_ = 5.743, *p* = 0.025; treatment × phenotype: *F*_(1_, _24)_ = 7.671, *p* = 0.011. Tukey multiple comparison test, *p < 0.05 vs. NN-vehicle; ^#^*p* < 0.05 vs. NN-nesfatin-1. **(C)** Parvocellular PVN (pPVN). **(D)** Magnocellular PVN (mPVN). **(E)** SON. **(F)** LHA. Two-way ANOVA, Means ± SEM, *n* = 7.

In order to identify hypothalamic nuclei, where nesfatin-1 resistance develops in PR rats, we measured the effect of icv nesfatin-1 on the number of fasting-activated neurons in the SON, PVN, ARC, and LHA (Figure 5). Treatment with nesfatin-1 significantly reduced the number of cFos-positive cells in the ARC of NN animals, but did not have this effect in PR rats (Figures 5A,B). No differences were found between the groups in the other nuclei investigated (Figures 5C–F).

### Chronic Intracerebroventricular Nesfatin-1 Treatment Improves the Glucose Homeostasis Only in Normal Nourished Rats

**FIGURE 6:**
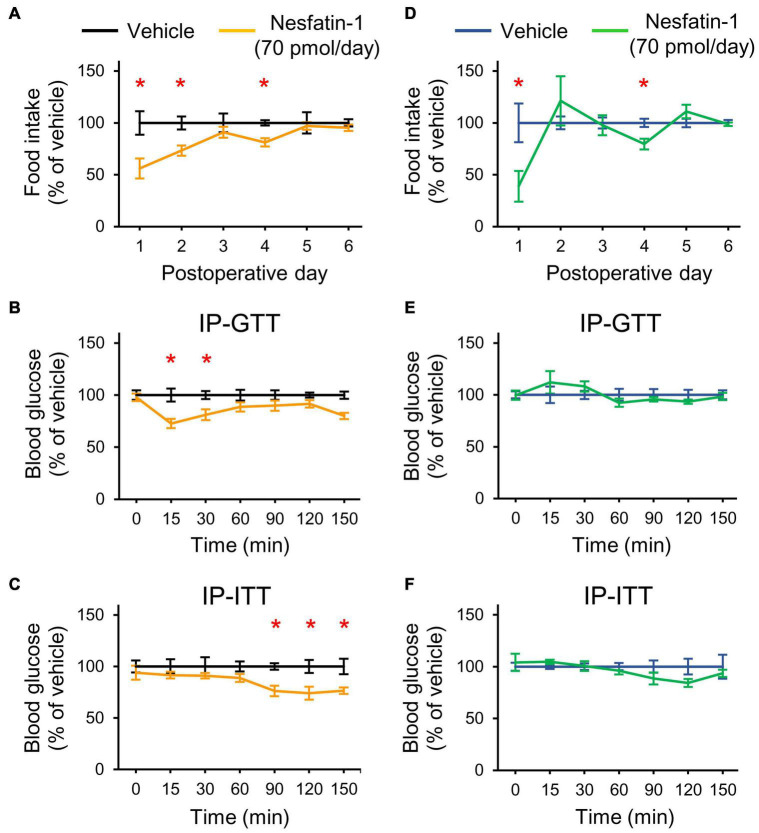
Effects of chronic icv nesfatin-1 (70 pmol/day) or vehicle infusions on the food intake and glucose homeostasis of NN and PR rats. Day zero was the day of cannula and infusion pump implantations as well as the beginning of the treatments. The tests were performed at the end of the treatment period (7 days). **(A)** Relative food intake of NN rats. Student’s *t*-test, vehicle vs. nesfatin-1, day 1: *p = 0.013, day 2: *p = 0.007, day 4: *p = 0.003. **(B)** IP-GTT shows increased glucose tolerance of NN-nesfatin-1 rats. Two-way RM ANOVA, effect of phenotype: *F*_(1_, _60)_ = 17.554, *p* = 0.002, effect of time: *F*_(6_, _60)_ = 81.649, *p* < 0.001, effect of phenotype × time: *F*_(6_, _60)_ = 3.760, *p* = 0.003. **(C)** IP-ITT shows increased insulin sensitivity. Two-way RM ANOVA, effect of phenotype: *F*_(1_, _60)_ = 19.324, *p* = 0.001, effect of time: *F*_(6_, _60)_ = 23.382, *p* < 0.001. Tukey multiple comparison tests, vehicle vs. nesfatin-1 within day *p < 0.05 for **(B,C)**. **(D)** Relative food intake of PR rats. Student’s *t*-test, vehicle vs. nesfatin-1, day1: *p = 0.029, day4: *p = 0.011 **(E,F)** IP-GTT and IP-ITT data of PR rats, respectively. No significant differences were seen. Means ± SEM, *n* = 6 for all graphs.

Finally, we assessed the effect of nesfatin-1 on glucose homeostasis. Since acute icv injection of 25 pmol nesfatin-1 was reported to have no effect on blood glucose levels in mice (Su et al., 2010), we applied chronic icv nesfatin-1 treatment (70 pmol/day, for 7 days). In NN rats, the treatment hindered the recovery from the surgery (bodyweight change_days 0–6_: −1 ± 3.2 g and −14.8 ± 2.4 g in the vehicle and nesfatin-1 treated groups, respectively, Student’s *t*-test, *p* = 0.034). Nesfatin-1 also reduced the food intake of NN animals (Figure 6A). The IP-GTT (Figure 6B) and IP-ITT (Figure 6C) performed at the end of the treatments showed an improved glucose tolerance and insulin sensitivity of nesfatin-1 treated NN rats relative to the vehicle-treated NN group.

Chronic treatment with nesfatin-1 did not induce nesfatin-1 resistance in NN animals, and even had a beneficial effect on their glucose homeostasis. We, therefore tested PR animals in a separate experiment. Treatment with nesfatin-1 did not affect the recovery of PR rats (bodyweight change_days 0–6_: 2.2 ± 2.1 and 5.3 ± 4.8 g in the vehicle and nesfatin-1 treated groups, respectively, Student’s *t*-test, *p* = 0.557), although slightly reduced their food intake (Figure 6D). Glucose homeostasis of PR rats was not affected by the treatment (Figures 6E,F).

## Discussion

The present study shows that in non-obese PR rats, central nesfatin-1 resistance probably contributes to the development of T2DM. Dysfunction of nesfatin-1 signaling in the ARC seems to play an important role in this process.

Insulin resistance and elevated hepatic glucose production are the main characteristics in T2DM. Impaired glucose tolerance and fasting hyperglycemia, which developed in PR rats with normal body composition between the 6th and 12th postnatal weeks, are among the early signs of the disease (Al-Goblan et al., 2014). Therefore, fetal programming by intrauterine protein restriction, predisposes development of T2DM as a primary effect, not secondarily to obesity.

According to the thrifty phenotype hypothesis (Barker et al., 1993), PR induces a predictive adaptive response of the organism by alternative metabolic programming of the organs to use the nutritional supply with a maximal effectiveness. In nutrient-rich environment, PR rats exhibited the typical signs of the thrifty phenotype; intense catch-up growth and preference for HFD relative to control peers (Langley-Evans et al., 2005). Importantly, preference for HFD developed after the completion of the catch up growth. This suggests that during the manifestation of the PR phenotype different mechanisms are turned on sequentially to modify the energy homeostasis.

Both adult PR and NN rats preferred HFD over ND, and caloric overconsumption was counter-regulated in both phenotypes with similar dynamics (after 3 days). However, PR animals consequently (from the 2nd day of the preference test) covered more of their daily caloric needs from HFD compared to control rats. In turn, they consumed less of ND to keep their daily caloric intake at normal level. Thus, energy balance regulation was functional in PR rats, but worked with different settings compared to NN animals.

Although the mRNA expression of NUCB2/nesfatin-1 was similar in the hypothalamus of PR and NN rats at birth, it was upregulated in the PVN, LHA and ARC in adult PR rats. Peripheral (blood) nesfatin-1 levels were unaltered. Despite chronic icv nesfatin-1 treatment causes weight loss (Oh-I et al., 2006), and acute injection of nesfatin-1 into the PVN or LHA reduces the cumulative food intake of rats (Chen X. et al., 2012), the increased nesfatin-1 production in PR rats was without a negative effect on food intake and bodyweight. Additionally, when we injected nesfatin-1 icv to rats, the treatment was not effective in reducing the nocturnal food intake of PR animals. Thus, adult PR rats were resistant to centrally added nesfatin-1, and NUCB2/nesfatin-1 mRNA was upregulated in the measured nuclei as compensation. Our data indicate that nesfatin-1 has an essential role in the regulation of the energy homeostasis *via* the PVN, LHA and ARC.

Nesfatin-1 reduced the number of fasting activated ARC neurons in NN rats, when it was applied icv at the time of the expected meal. Nesfatin-1 applied icv also reduces the food intake in previously fasted rats (Yosten and Samson, 2009). The phenotype of non-responding cells would be hard to identify. However, the neuronal activity in two key cell populations of the ARC correlates with energy status in rodents. Orexigenic, neuropeptide Y (NPY) producing ARC neurons are activated by fasting, while anorexigenic, melanocortin expressing neurons are activated by refeeding (Fekete et al., 2012; Betley et al., 2013). Almost all (∼94%) of activated, i.e., cFos-immunoreactive neurons are NPY cells in the ARC of fasted mice (Becskei et al., 2009). Therefore, one explanation is that nesfatin-1 reduced the number of cFos-immunopositive cells by acting on NPY neurons. This would be consistent with *ex vivo* data showing that nesfatin-1 hyperpolarizes NPY cells (Price et al., 2008). Regardless of this, fasting triggers an extensive orexigenic drive primarily *via* the activation of the NPY cells in the ARC. Nesfatin-1 reduced the orexigenic drive (i.e., food intake) in NN, but not in PR animals, and our cFos data point to the ARC as a site of action. The ARC plays a master role in the control of food intake, energy expenditure and glucose homeostasis by integrating both central and peripheral/neuronal and humoral (leptin, insulin, glucose, ghrelin) inputs (Timper and Brüning, 2017). The inhibition of the orexigenic drive in the ARC by nesfatin-1 and the impairment of this function in PR animals is therefore of great relevance for understanding the role of nesfatin-1 in the regulation of the energy homeostasis. It also identifies the ARC as a site, where nesfatin-1 resistance in PR animals most probably has functional consequences.

The hypothalamic centers for food intake and blood glucose level regulation overlap (Kleinridders et al., 2009; Sandoval et al., 2009). We provided a direct evidence that hypothalamic nesfatin-1 resistance affects food intake, and may contribute to deterioration of the glucose homeostasis in PR rats. Hepatic glucose flux increases, while glucose uptake of peripheral tissues decreases in mice with hypothalamic knockdown of NUCB2/nesfatin-1 (Wu et al., 2014). Nesfatin-1 injected icv increases muscle glucose absorption and inhibits hepatic glucose production in rats during euglycemic-hyperinsulinemic clamping (Yang et al., 2012). Now, we showed that chronic icv nesfatin-1 treatment improves the performance of NN rats in IP-GTT and IP-ITT tests. The treatment at the dose and timing used here was ineffective in PR rats.

The limitation of our study is that the exact mechanism of how T2DM develops in hypothalamic nesfatin-1 resistance remains to be elucidated. Unfortunately, nesfatin-1 agonists and antagonists are not available, since the receptor of nesfatin-1 is unidentified. Transgenic mouse models may help future studies. As nesfatin-1 is not transported to axons, local, auto/paracrine mechanisms of nesfatin-1’s action are assumed *via* dendritic release (Brailoiu et al., 2007; Foo et al., 2008). Indeed, neurogenesis of nesfatin-1 cells was complete, but the time course of cell formation was disturbed in PR rats, suggesting that hypothalamic nesfatin-1 resistance in PR rats may have developed due to alternative fetal programming of hypothalamic nesfatin-1 neurons. The importance of the ARC is stressed, since both POMC and NPY neurons in the ARC were shown to regulate glucose production of the liver (Könner et al., 2007; Dodd et al., 2018). Nesfatin-1 is abundandly expressed in the ARC (Brailoiu et al., 2007), thus, it may act on the glucose homeostasis by influencing the function of these neurons. Indeed, most of the nesfatin-1’s action requires melanocortin 3/4 receptor, and nesfatin-1 inhibits the NPY neurons *in vitro* (Schalla et al., 2020).

In summary, we demonstrated the existence of hypothalamic nesfatin-1 resistance in PR rats by using two different approaches. Our data suggest that hypothalamic nesfatin-1 resistance may be a substantial factor in development of non-obese T2DM induced by IUGR. Our finding, that treatment with nesfatin-1 may improve the glucose homeostasis and that nesfatin-1 acts independently of leptin (Shimizu et al., 2009), makes the hypothalamic nesfatin-1 system a potential therapeutic target for the treatment of T2DM.

## Data Availability Statement

The raw data supporting the conclusions of this article will be made available by the authors, without undue reservation.

## Ethics Statement

The animal study was reviewed and approved by the Semmelweis University Workplace Animal Welfare Body Committee Semmelweis University, Budapest, Üllői út 26., Hungary.

## Author Contributions

ZT designed the research. MD, KK, KO, KS, and ZT performed the experiments and analyzed the data. PV performed molecular biology. AS-S performed MRI. MD, KK, CF, and ZT wrote the manuscript. All authors contributed to the article and approved the submitted version.

## Conflict of Interest

The authors declare that the research was conducted in the absence of any commercial or financial relationships that could be construed as a potential conflict of interest.

## Publisher’s Note

All claims expressed in this article are solely those of the authors and do not necessarily represent those of their affiliated organizations, or those of the publisher, the editors and the reviewers. Any product that may be evaluated in this article, or claim that may be made by its manufacturer, is not guaranteed or endorsed by the publisher.

## Footnotes

1
https://blast.ncbi.nlm.nih.gov/Blast.cgi
